# Spontaneous Trabeculectomy Bleb Reformation and Regain of Function Following Phacoemulsification

**DOI:** 10.7759/cureus.16979

**Published:** 2021-08-07

**Authors:** Wassef Chanbour, Hani Chanbour, Karim F Tomey, Ziad Khoueir

**Affiliations:** 1 Ophthalmology, Beirut Eye & ENT Specialist Hospital, Beirut, LBN; 2 Medicine, Lebanese University Faculty of Medicine, Beirut, LBN

**Keywords:** phacoemulsification, trabeculectomy, bleb reformation, glaucoma, cataract

## Abstract

Phacoemulsification and cataract surgery in general often lead to trabeculectomy bleb failure. We herein describe an unusual occurrence in a 79-year-old female who had a failed trabeculectomy bleb for one year prior to presentation, and whose failed bleb became reformed, and she regained function on the first day post-phacoemulsification, manifesting as a decrease in intraocular pressure. Topical corticosteroids were used for one month postoperatively and the bleb remained functional over more than six months of follow-up. It is most likely that the intraocular pressure elevation that occurs during phacoemulsification was responsible for the reformation of the bleb, even after having been a failed bleb for a whole year.

## Introduction

Trabeculectomy remains the most commonly performed glaucoma surgery to control intraocular pressure (IOP) [1]. Cataracts are known to progress faster following this surgery [2-3], and hence many glaucomatous eyes with blebs will require cataract surgery at some stage.

Multiple studies have shown that performing cataract surgery in an eye with a functioning filtering bleb can result in bleb failure and IOP elevation [4-5] while other studies reported that phacoemulsification had no effect on the bleb function or the IOP [6-7].

The ocular inflammatory reaction post phacoemulsification has been proposed to be the cause of bleb failure through promoting conjunctival scarring, and this is true especially during the first year following trabeculectomy. In contrast, performing phacoemulsification at a later time has been found to be less likely to have an adverse effect on the bleb, and it was not a risk factor for further increase in IOP [8].

We herein describe a case of spontaneous old filtration bleb reopening following cataract surgery.

## Case presentation

A 79-year-old female, who had previously undergone a trabeculectomy 15 years earlier in her right eye for primary open-angle glaucoma, presented with progressive decline in her visual acuity over one year, from 20/25 to 20/50. She was found to have a 2+ nuclear cataract and an absent bleb, although it had been present one year earlier.

Intraocular pressure in that eye on a timolol/dorzoalmide (6.8 mg/22.3 mg per mL) combination, brimonidine (0.02%), and travoprost (0.004%) was 10 mmHg.

Under peri-bulbar anesthesia, and through a 3.0-mm clear-corneal incision at 11 o’clock, non-complicated phacoemulsification with in-the-bag foldable intraocular lens implantation was performed on the right eye. A tobramycin 0.3% and dexamethasone 0.1% combination was started postoperatively four times per day, to be tapered during the following month.

**Figure 1:**
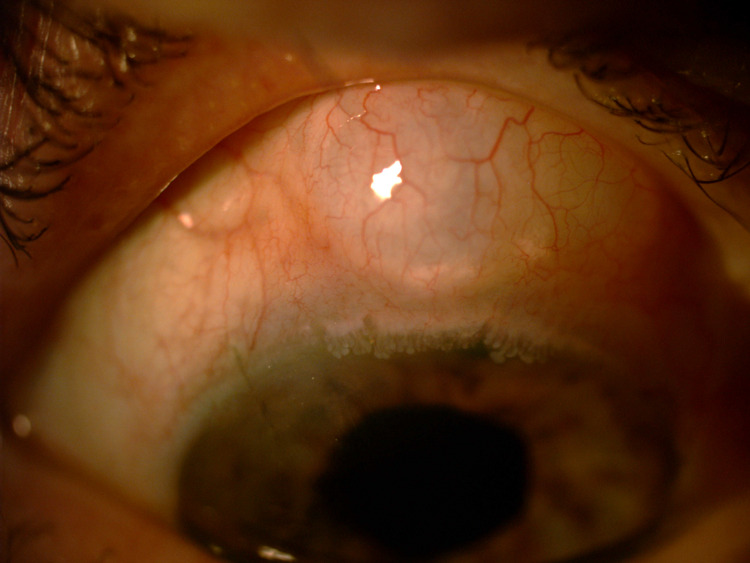
Day 1 post-phacoemulsification photo showing the encapsulated bleb

**Figure 2:**
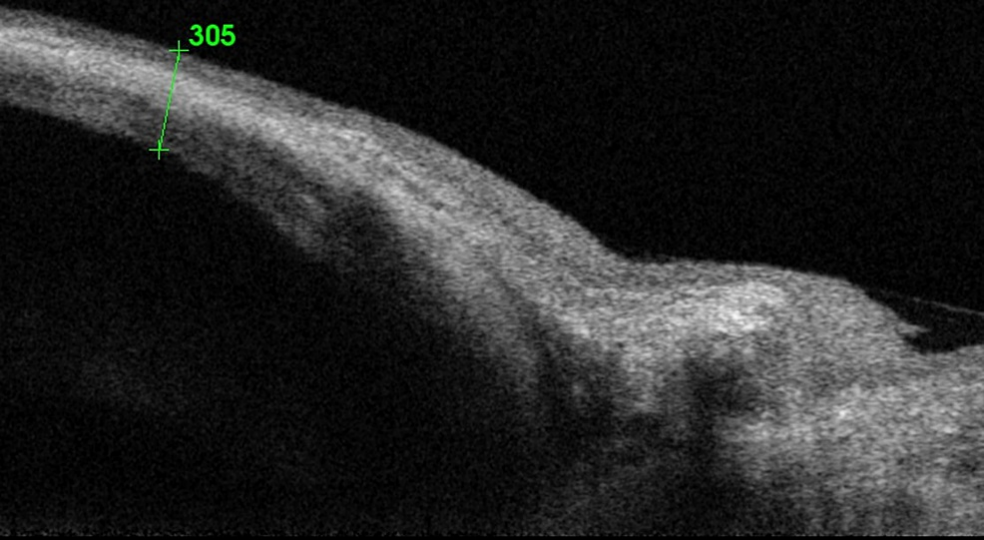
OCT showing the elevated bleb with subconjunctival fluid with a conjunctival thickness of 305 µm OCT: optical coherence tomography

**Figure 3:**
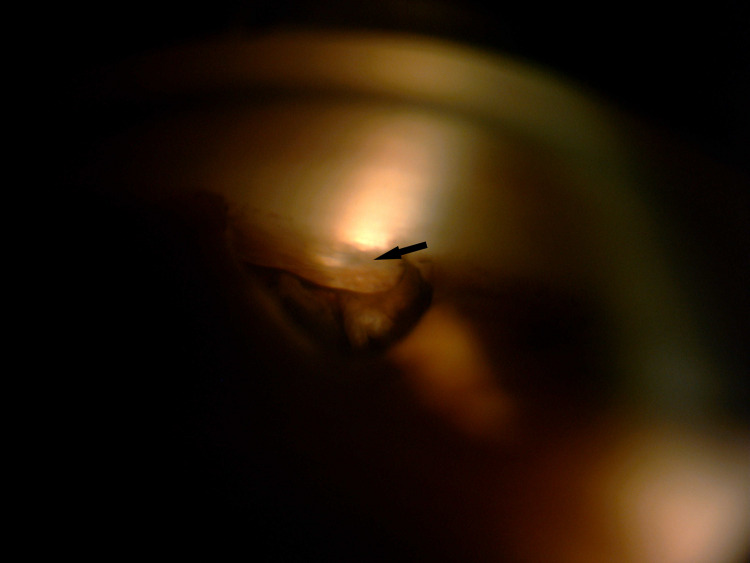
Gonioscopy showing the surgical iridectomy and the patent internal ostium of the trabeculectomy (arrow)

On the first postoperative day, the patient presented with a pressure of 6 mmHg and an elevated and formed bleb, which was encapsulated (Figures 1-2). On gonioscopy, the internal trabeculectomy ostium was found to be patent (Figure 3). All the topical anti-glaucoma drops were stopped.

The follow-up was uneventful. Six months later, the IOP stabilized at 10 mmHg on travoprost only. The bleb was still present and the final uncorrected visual acuity was 20/20 in the operated eye.

## Discussion

To our knowledge, this is the first case to be reported of a failed trabeculectomy filtration bleb spontaneously reforming and regaining its function following phacoemulsification.

The most probable explanation is that the event occurred during the surgery. Khng et al. proved that IOP could reach 60 mmHg during coaxial and bimanual phacoemulsification [9]. We theorize that the high intraoperative intraocular pressure reopened the trabeculectomy flap (sort of an ab interno revision), leading to a decrease in the postoperative IOP to the extent of slight hypotony.

Iatrogenic bleb formation following cataract surgery has been reported previously. It occurs if the wound leaks under a sealed conjunctiva post-cataract surgery, even in eyes that had not previously undergone trabeculectomy [10-11]. In our case, the corneal incision was located away from the trabeculectomy flap and the bleb location. Also, the corneal incision was sutured with 10-0 nylon. That is why the possibility of an inadvertent filtering bleb caused by the incision was eliminated.

The newly formed bleb was encapsulated but functional, and thus the significant decrease in IOP obviated the use of some glaucoma medications. We believe also that the continuous use of potent topical corticosteroids following the cataract surgery decreased the postoperative inflammation, thus preventing the new bleb from scarring. The use of topical corticosteroids in the early postoperative period after trabeculectomy has been found to be efficient in stabilizing the IOP and visual function in patients with progressive glaucomatous disease [12]. In a study conducted by Klink J et al., 13.3% of the trabeculectomy patients had a decreased IOP of more than 2 mmHg post phacoemulsification, but it was not associated with an improved bleb morphology [13].

In brief, this case illustrates that bleb reformation and regain of function can occur, though rarely, following phacoemulsification and even after the trabeculectomy had failed for a long time, one year in our case. Topical steroids should be used in the early postoperative period in order to maintain bleb function.

## Conclusions

Our case demonstrates that not all cataract surgeries have ill effects on eyes previously operated on for glaucoma. The bleb can be reformed and the trabeculectomy flap reopened even one year after having been scarred, and this is conceivably caused by the bouts of severe IOP elevation that usually occur during the course of the phacoemulsification procedure.
